# Predictive modeling for metastasis in oncology: current methods and future directions

**DOI:** 10.1097/MS9.0000000000003279

**Published:** 2025-05-21

**Authors:** Ghulam H. Abbas, Edmon R. Khouri, Omar Thaher, Safwan Taha, Miljana Vladimirov, Rodolfo J. Oviedo, Jeremias Schmidt, Dirk Bausch, Sjaak Pouwels

**Affiliations:** a Faculty of Medicine, Ala-Too International University, Bishkek, Kyrgyz Republic; b Department of Medicine, Mass General Brigham, Boston, Massachusetts, USA; c School of Medicine, University of Jordan, Amman, Jordan; d Department of Surgery, Marien Hospital Herne, University Hospital of Ruhr University Bochum, Herne, NRW, Germany; e The Metabolic and Bariatric Surgery Center of Excellence (SRC), Mediclinic Airport Road Hospital, Abu Dhabi, UAE; f Department of Surgery, Bielefeld University Campus Detmold, Klinikum Lippe, Detmold, NRW, Germany; g Nacogdoches Medical Center, Nacogdoches, Texas, USA; h University of Houston Tilman J. Fertitta Family College of Medicine, Houston, Texas, USA; i Sam Houston State University College of Osteopathic Medicine, Conroe, Texas, USA; j Department of Plastic, Reconstructive and Aesthetic Surgery, Helios Klinikum Berlin-Buch, Berlin, Germany; k Department of Intensive Care Medicine, Elisabeth-Tweesteden Hospital Tilburg, The Netherlands

**Keywords:** artificial intelligence, cancer metastasis, cancer progression, clinical oncology, machine learning, metastasis prediction, oncology biomarkers, predictive modeling, risk prediction

## Abstract

Predictive modeling for metastasis in oncology has gained significant traction due to its potential to improve prognosis, guide treatment strategies and enhance patient outcomes. Current methods leverage advancements in machine learning, genomics and imaging technologies to predict the likelihood of cancer spread. Techniques such as logistic regression, decision trees, support vector machines and neural networks have been employed to analyze clinical, pathological, and molecular data. Genomic profiling, liquid biopsies, and radiomics are increasingly integrated into these models to identify metastatic patterns and risk factors. Despite these advances, challenges persist, including data heterogeneity, model interpretability, and the need for larger, high-quality datasets for validation. Furthermore, the integration of artificial intelligence with precision medicine offers promising avenues for more personalized metastasis predictions. Future directions focus on enhancing model accuracy through deep learning, improving the interpretability of black-box models, and incorporating multi-omics data to capture the complexity of metastatic mechanisms. With the advent of advanced computational tools and growing datasets, predictive modeling in oncology is poised to revolutionize metastasis management, offering clinicians’ valuable insights for early detection and tailored treatment strategies.

## Introduction

Cancer remains one of the most challenging health issues worldwide, with metastasis being a critical factor in patient outcomes. The spread of cancer cells from the primary tumor to distant organs has a significant impact on survival rates and treatment strategies. As a result, there is growing interest in developing predictive models for metastasis, particularly in the field of oncology. Machine learning (ML) and artificial intelligence (AI) are at the forefront of this endeavor, offering new ways to analyze complex biological data and identify patterns that may indicate a higher risk of metastatic spread^[1]^.

Recent advances in predictive modeling have led to improved methods for early detection and prognosis of cancer metastasis. These models use various data types, including genetic information, imaging results, and clinical biomarkers, to assess the likelihood of metastatic events. ML algorithms are particularly well suited to handle the large datasets involved in cancer research, allowing for more accurate predictions of lymph node metastasis and distant organ involvement^[^1^]^. As the field continues to evolve, researchers are exploring new technologies and integrating multiple data sources to enhance the accuracy and clinical usefulness of these predictive tools, with the ultimate goal of improving patient care and survival rates in oncology.

Cancer metastasis is a complex and critical process that has a significant impact on patient outcomes and presents substantial challenges in early detection and treatment. This section explores the definition and mechanisms of metastasis, its effects on patient prognosis, and the difficulties faced in identifying metastatic disease at an early stage^[^2^]^.

Metastasis is defined as the spread of cancer cells from the primary tumor to anatomically distinct sites within the body. This process involves a series of intricate steps known as the metastatic cascade. The cascade begins with the dissociation of cancer cells from the primary tumor, followed by their invasion of surrounding tissues and entry into the bloodstream or lymphatic system^[^3^]^. These circulating tumor cells must then survive in the hostile environment of the circulatory system, adhere to blood vessel walls at distant sites, extravasate into the new tissue, and finally establish a secondary tumor.

The metastatic process is highly inefficient, with only a small fraction of disseminated cells successfully forming clinically relevant metastases. Research has shown that tumors can shed millions of cells into the bloodstream daily, yet very few of these cells survive to colonize distant organs. This inefficiency is due to the numerous obstacles cancer cells must overcome at each step of the metastatic cascade.

Metastasis has a profound impact on patient outcomes and remains the leading cause of cancer-related deaths. Its presence drastically worsens prognosis and necessitates more aggressive treatment approaches. In fact, most cancer mortality is linked to metastasis, as patients with localized tumors typically have better survival rates. Evidence suggests that the metastatic process may begin early, with studies indicating that 60% to 70% of patients have already initiated metastasis at diagnosis. Even in cases of small, node-negative tumors (T1N0), the risk of developing distant metastases can be as high as 15% to 25%^[^4^]^. Despite these grim statistics, survival rates have improved in some patient subgroups, particularly with the advent of metastasis-directed therapies for those with low metastatic burdens.

However, early detection of metastatic disease remains a challenge due to limitations in current diagnostic techniques, which often fail to identify micrometastases or disseminated tumor cells (DTCs). The variability in latency periods between initial diagnosis and metastatic recurrence further complicates timely detection, as some cancer cells can remain dormant for years before reactivating. The intricate interactions between disseminated tumor cells and the microenvironment, along with host and environmental factors, add additional layers of complexity to metastasis prediction. Moreover, screening methods are affected by biases, such as lead-time and overdiagnosis of indolent cancers. Addressing these challenges is essential for improving early detection and management of metastatic disease, which could potentially enhance outcomes for cancer patients as research and new technologies evolve^[^5^]^.

**Figure 1.**
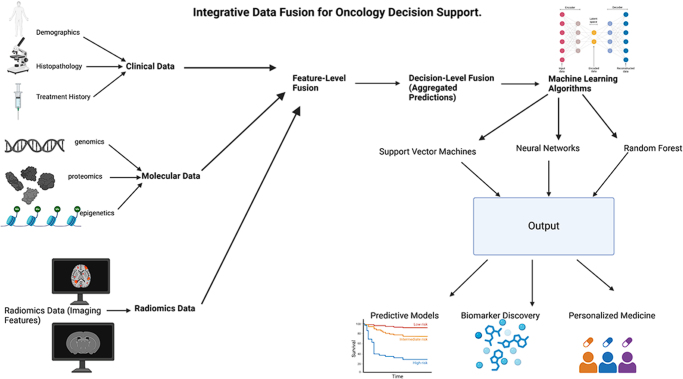
Integrative data fusion for oncology decision support^[^68-82^]^. This diagram illustrates the fusion of clinical, molecular, and radiomics data for oncology decision-making. It showcases feature- and decision-level fusion, applying machine learning algorithms (SVM, neural networks, and random forest) to generate outputs for predictive modeling, biomarker discovery, and personalized medicine.

This manuscript evaluates advancements in predictive oncology, emphasizing the transformative potential of integrating multi-omics, imaging, and clinical datasets. Integrating machine learning with clinical decision-making improves risk assessment in Fig. 1. These approaches aim to address the limitations of current diagnostic and predictive models, By contrasting traditional methodologies with emerging technologies, the manuscript highlights the benefits of multi-modal integration in enhancing predictive accuracy and enabling personalized oncology care. This integrated approach provides a framework for developing more effective predictive tools, ultimately improving early detection, treatment strategies, and patient outcomes. The discussion outlines a plan for translating these innovations into clinical applications, paving the way for a new era in cancer management.

## Current predictive models for metastasis

Predictive models for metastasis in oncology have evolved significantly, leveraging diverse data sources and computational methods to assess the risk of cancer spread. These models incorporate clinical, pathological, genomic, and imaging-based approaches to offer more precise predictions and informed treatment strategies. Researchers are increasingly focused on identifying key factors that influence metastasis, allowing for more tailored, patient-specific interventions. In particular, the integration of multi-omics data combining genomic, transcriptomic, and proteomic information alongside advanced imaging technologies like radiomics, is improving the ability of these models to predict the risk of metastasis and recurrence^[^6^]^.

**HIGHLIGHTS**

- Revolutionizing cancer prognosis: Predictive modeling for metastasis in oncology is transforming cancer prognosis by integrating AI, machine learning, and multi-omics data.
- Machine learning in metastasis prediction: Advanced ML techniques like decision trees, support vector machines (SVM), and neural networks improve the accuracy of predicting cancer spread.
- Liquid biopsies for early detection: Non-invasive liquid biopsies utilizing circulating tumor DNA (ctDNA) and circulating tumor cells (CTCs) provide real-time insights into metastasis.
- Radiomics and AI in imaging: AI-driven radiomics extracts quantitative imaging features that predict tumor aggressiveness and metastatic potential with high precision.
- Genomic biomarkers for personalized treatment: Specific genetic mutations (e.g., TP53, KRAS, and EGFR) help stratify patients and guide precision oncology approaches.
- Challenges in model interpretability: Despite AI’s predictive power, the “black-box” nature of deep learning models remains a major obstacle to clinical adoption.
- Multi-omics data fusion enhances accuracy: Combining genomic, transcriptomic, and proteomic data with imaging and clinical records results in superior metastasis prediction models.
- Explainable AI (XAI) for clinical trust: Emerging techniques in explainable AI (XAI) aim to make predictive models more transparent and interpretable for oncologists.
- Regulatory and clinical implementation hurdles: Validating AI-based metastasis models in real-world clinical settings requires overcoming regulatory, ethical, and data harmonization challenges.
- Future directions – AI & liquid biopsy convergence: The integration of AI with liquid biopsies and single-cell sequencing is set to redefine early metastasis detection and cancer management.

One of the most important areas of study in predictive modeling is identifying clinical and pathological factors that can forecast metastasis in various cancers. For instance, several studies have made significant strides in understanding the risk of lymph node metastasis (LNM) in non-small cell lung cancer (NSCLC). Yu *et al* identified tumor size, pleural invasion, and carcinoembryonic antigen (CEA) levels as independent risk factors for LNM, highlighting the need for these markers to be considered when planning treatment strategies^[^5^]^. Pani *et al* further discovered that certain histologic subtypes of NSCLC, such as adenocarcinoma with micropapillary and solid components, could be linked to lymph node status, providing additional insights into how tumor biology correlates with metastasis risk^[^5,6^]^. Both studies used univariate and multivariate analyses, demonstrating that combining multiple clinicopathological predictors can reveal patterns of metastasis that might be missed when using a single-factor approach^[^7^]^.

Histological details have proven particularly valuable in predicting metastasis risk. Tumor subtypes with solid, micropapillary and acinar components have been associated with a higher likelihood of LNM, whereas tumors with lepidic components are often associated with LNM-free disease. These findings emphasize the importance of including detailed histological features in intraoperative pathology reports. Such detailed reporting is especially useful in cases where surgeons must decide between performing a lobectomy or sublobar resection, balancing the need for tumor removal with the goal of preserving as much healthy lung tissue as possible^[^8^]^.

In addition to clinical and pathological factors, the growing field of genomics is reshaping how metastasis is understood and predicted. Genomic profiling of tumors has revealed specific mutations and molecular alterations that drive metastasis, including mutations in genes such as TP53, KRAS and EGFR, which have been linked to more aggressive tumor behavior and a higher risk of metastasis in several cancer types^[^9^]^. Liquid biopsies, which analyze circulating tumor DNA (ctDNA) or circulating tumor cells (CTCs) in blood samples, are emerging as non-invasive tools that can monitor tumor evolution in real-time and potentially detect metastasis before it becomes clinically evident^[^10^]^. These biomarkers offer a dynamic approach to tracking tumor progression, which could allow for earlier intervention and better-tailored treatment strategies^[^9,10^]^.

Imaging techniques are also playing a key role in predictive modeling for metastasis. Radiomics, the extraction of quantitative features from medical images using artificial intelligence (AI), is enhancing the ability of models to predict tumor behavior and metastatic potential. By analyzing patterns that may not be visible to the human eye, radiomics can provide additional predictive power when combined with clinical and pathological data^[^11^]^. This approach has shown promise in identifying subtle features of tumors that correlate with a higher likelihood of metastasis, particularly in cancers such as lung, breast and prostate cancer.

Despite these advances, challenges remain in creating universally applicable predictive models for metastasis. One major hurdle is the heterogeneity of cancer, as tumors of the same type can behave very differently based on their molecular and environmental context. Additionally, while large datasets are crucial for training robust predictive models, the quality and consistency of these datasets can vary, leading to potential limitations in the generalizability of the models. The interpretability of AI-based models also remains a challenge, as many deep learning algorithms function as “black boxes” providing predictions without clear explanations of how they were derived^[^12^]^. This lack of transparency can make clinicians hesitant to rely solely on AI-generated predictions, highlighting the need for interpretable ML techniques in clinical practice.

Future directions in predictive modeling for metastasis will likely involve a more integrated approach, combining multi-omics data, advanced imaging techniques, and AI to create more accurate and clinically actionable models. The incorporation of real-world data from electronic health records, along with continuous updates based on patient outcomes, will further refine these models, allowing for ongoing improvements in their predictive power. Personalized medicine, where treatment decisions are tailored to the individuals based on their unique tumor profile, is becoming increasingly feasible as predictive models become more sophisticated and data-driven. These advances hold the promise of improving early detection of metastasis, optimizing treatment strategies, and ultimately enhancing survival rates for cancer patients^[^13^]^.

Predictive models and their limitations are summarized in Table 1, the table maps specific studies to their respective methodologies, applications, and challenges.

**Table 1:** Introductory approaches, techniques, applications, methods and limitations in predictive modeling for metastasis in oncology^[^1-24^]^

Approach	Key factors/techniques	Examples/applications	Methods used	Challenges/limitations	References
Clinical/pathological	Tumor size, histologic subtypes, CEA levels	LNM prediction in thyroid cancer (Liu *et al*, 2024) Prognostic factors in renal cell carcinoma (Sun *et al*, 2011) Histologic subtypes in NSCLC (Pani *et al*)	Meta-analysis, multivariate analysis	Requires detailed histopathology; variability in tumor biology	^[^6,7^]^
Machine learning/nomograms	Predictive algorithms, nomograms	Oral cavity cancer outcomes (Adeoye *et al*, 2021) Prostate cancer LNM prediction (Wang *et al*, 2023)	Machine learning (ML), systematic reviews	Model interpretability (“black box” issue); dataset heterogeneity	^[^8,13^]^
Genomics	Mutations (TP53, KRAS), liquid biopsies (ctDNA)	Endometrial cancer LNM models (Li *et al*, 2024) Renal cell carcinoma management (Lee *et al*, 2012)	Genomic profiling, systematic reviews	Tumor heterogeneity; validation in diverse cohorts	^[^9,10^]^
Imaging/radiomics	Radiomics, AI-based imaging analysis	Prostate cancer radiotherapy outcomes (Roach *et al*, 2009)	Radiomic feature extraction	Limited interpretability of imaging patterns; dataset quality	^[^12^]^
Multi-omics integration	Machine learning with omics data (genomic, proteomic)	Metastasis prediction using omics (Albaradei *et al*, 2021)	Deep learning, ML pipelines	Data integration complexity; generalizability across cancer types	^[^11^]^

## Genomic and molecular biomarkers

Genomic and molecular biomarkers have emerged as essential tools for predicting metastasis and guiding personalized treatment decisions in oncology. As cancer research advances, understanding the molecular underpinnings of cancer spread has led to the discovery of key genetic, epigenetic, and molecular signatures associated with metastatic potential. These biomarkers provide a more individualized approach to patient care, improving prognosis accuracy and informing more targeted therapies. In breast cancer, for example, a study identified a 13-gene profile associated with early (≤36 months) symptomatic brain metastasis. This profile was further refined to a highly predictive 3-gene classifier (RAD51, HDGF and TPR), underscoring the ability of genomics to pinpoint specific molecular drivers of metastatic spread^[^13^]^. Such findings are critical for early intervention and can lead to novel therapeutic approaches aimed at preventing or delaying metastasis.

Germline genetic markers have also gained prominence in predicting metastasis and guiding treatment. Traditionally, germline mutations were primarily associated with cancer susceptibility. However, these mutations are now recognized as important prognostic and predictive markers for tailored therapies. For example, poly (ADP-ribose) polymerase (PARP) inhibitors have proven highly effective in treating breast and ovarian cancers with germline BRCA mutations^[^14^]^. BRCA1 and BRCA2 mutations impair DNA repair mechanisms, making tumor cells particularly vulnerable to PARP inhibitors, which further inhibit the repair of damaged DNA, leading to cancer cell death^[^14^]^. This approach exemplifies the growing field of synthetic lethality, where specific genetic alterations in cancer cells are targeted to exploit their vulnerabilities^[^15^]^. Beyond BRCA mutations, other germline markers are being explored for their potential to predict treatment responses and metastatic risk across various cancer types, including prostate and pancreatic cancers.

In addition to germline mutations, somatic alterations in tumors play a critical role in metastasis prediction. Advances in next-generation sequencing (NGS) have made it possible to detect somatic mutations, gene amplifications and copy number variations that contribute to cancer progression. For instance, mutations in the TP53, KRAS and EGFR genes are commonly associated with aggressive tumor behavior and a higher likelihood of metastasis in cancers such as lung, colorectal and head and neck cancers^[^16^]^. These mutations are not only prognostic indicators but also therapeutic targets, with drugs such as EGFR inhibitors and KRAS-targeted therapies showing promise in patients with specific mutational profiles. The ability to profile a tumor’s genetic landscape through NGS allows for more precise predictions of metastatic potential and the development of personalized treatment plans.

MicroRNAs (miRNAs) have also gained attention as promising biomarkers for cancer progression and metastasis. miRNAs are small non-coding RNA molecules that regulate gene expression post-transcriptionally. Their role in cancer has been extensively studied, and several miRNAs have been identified as key players in oncogenesis and metastasis. Some miRNAs, such as miR-21 and miR-155, are overexpressed in many cancers and function as oncogenes by promoting cell proliferation, invasion and metastasis. These oncogenic miRNAs can activate pro-metastatic pathways, making them valuable biomarkers for predicting metastasis. Conversely, other miRNAs, like let-7, miR-128b, miR-15 and miR-16, are tumor suppressors that are often downregulated in cancers^[^17^]^. Their loss contributes to the progression and spread of cancer by allowing tumor cells to escape normal growth control mechanisms.

One of the most promising aspects of miRNA research is their potential for use in liquid biopsy-based diagnostics. miRNAs with differential expression in tumor tissues can also be detected in circulating tumor RNA (ctRNA) in body fluids such as blood and urine^[^18^]^. This makes miRNAs attractive candidates for non-invasive cancer biomarkers. Liquid biopsies, which analyze ctRNA, circulating tumor DNA (ctDNA) and circulating tumor cells (CTCs), offer a less invasive alternative to traditional tissue biopsies and allow for real-time monitoring of tumor dynamics^[^18^]^. Liquid biopsies can provide valuable information about tumor heterogeneity, the emergence of resistance mutations and the risk of metastasis, all of which are critical for optimizing treatment plans. In particular, miRNAs circulating in the bloodstream have been shown to reflect the metastatic status of tumors, making them useful for early detection and monitoring disease progression^[^19^]^.

**Figure 2.**
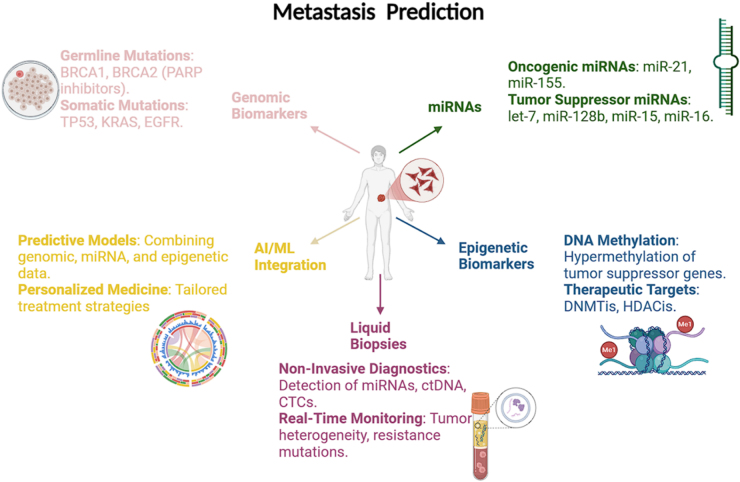
Metastasis prediction framework^[^21-29^]^. Visualizes key biomarkers and technologies in predicting cancer metastasis. It highlights the role of genomic, epigenetic, and miRNA biomarkers, the use of liquid biopsies for non-invasive diagnostics, and AI/ML integration for predictive modeling and Personalized Medicine.

The clinical utility of miRNAs as metastasis biomarkers extends beyond their diagnostic potential. miRNAs are also being explored as therapeutic targets. Given their role in regulating key oncogenic and tumor suppressive pathways, modulating miRNA expression could represent a novel approach to treating metastatic cancer. For example, anti-miR therapies aim to inhibit the function of oncogenic miRNAs, while miRNA mimics can restore the expression of tumor suppressor miRNAs. These therapeutic strategies are still in the experimental stage, but they hold great promise for improving outcomes in patients with metastatic disease. The metastasis prediction framework is illustrated in Fig. 2.

Another exciting development in the field of metastasis prediction is the use of epigenetic biomarkers. Epigenetic alterations, such as DNA methylation, histone modifications and chromatin remodeling, play a crucial role in regulating gene expression and can influence the metastatic potential of tumors. Aberrant DNA methylation patterns, for example, have been linked to the silencing of tumor suppressor genes and the activation of metastasis-promoting genes^[^19^]^. Hypermethylation of specific gene promoters has been associated with poor prognosis and an increased risk of metastasis in cancers such as colorectal, lung and breast cancers^[^20^]^. The reversibility of epigenetic changes makes them attractive targets for therapeutic intervention, with drugs such as DNA methyltransferase inhibitors (DNMTis) and histone deacetylase inhibitors (HDACis) showing potential in clinical trials for reactivating silenced tumor suppressor genes and reducing metastasis.

The integration of genomic, epigenetic and molecular biomarkers into predictive models for metastasis is revolutionizing cancer care. These models are becoming increasingly sophisticated, combining data from multiple sources to provide a more comprehensive view of a tumor’s metastatic potential. AI and ML algorithms are being employed to analyze complex datasets and identify patterns that predict metastasis^[^21^]^. By integrating molecular biomarkers, clinical data and imaging results, these models are improving the accuracy of metastasis prediction and enabling more personalized treatment approaches. As research in this area continues to advance, the development of highly accurate, non-invasive biomarkers for predicting metastasis will play a critical role in improving cancer outcomes and reducing the burden of metastatic disease. Several miRNAs play crucial roles in cancer progression and metastasis, as detailed in Table 2.

**Table 2:** Functional roles of key miRNAs across medical fields^[^29^-^46^]^

Field	Applications	Key miRNAs	Description	References on miRNA applications
Oncology	Diagnosis, prognosis, and treatment of various cancers	miR-21, miR-155, miR-34a	miR-21 promotes tumor growth, miR-155 is linked to lymphoma and breast cancer, and miR-34a acts as a tumor suppressor.	Calin, G. A., & Croce, C. M. (2006). MicroRNA signatures in human cancers. *Nature Reviews Cancer, 6*(11), 857–866. https://doi.org/10.1038/nrc1997
				Garzon, R., Calin, G. A., & Croce, C. M. (2009). MicroRNAs in cancer. *Annual Review of Medicine, 60*, 167–179. https://doi.org/10.1146/annurev.med.59.053006.104707
				Misso, G., Di Martino, M. T., De Rosa, G., Farooqi, A. A., Lombardi, A., Campani, V., .. & Caraglia, M. (2014). Mir-34: A new weapon against cancer? *Molecular Therapy – Nucleic Acids, 3*, e194. https://doi.org/10.1038/mtna.2014.47
Cardiovascular diseases	Biomarkers for heart failure, atherosclerosis, and myocardial infarction	miR-1, miR-133, miR-208	miR-1 and miR-133 are involved in cardiac hypertrophy and arrhythmias, while miR-208 regulates stress-induced cardiac remodeling.	van Rooij, E., Sutherland, L. B., Qi, X., Richardson, J. A., Hill, J., & Olson, E. N. (2006). Control of stress-dependent cardiac growth and gene expression by a microRNA. *Science, 316*(5824), 575–579. https://doi.org/10.1126/science.1139089
				Small, E. M., & Olson, E. N. (2010). Pervasive roles of microRNAs in cardiovascular biology. *Nature, 469*(7330), 336–342. https://doi.org/10.1038/nature09790
				Condorelli, G., Latronico, M. V., & Cavarretta, E. (2014). microRNAs in cardiovascular diseases: Current knowledge and the road ahead. *Journal of the American College of Cardiology, 63*(21), 2177–2187. https://doi.org/10.1016/j.jacc.2014.01.050
Neurodegenerative diseases	Biomarkers and therapeutic targets for Alzheimer’s, Parkinson’s, and ALS	miR-9, miR-132, miR-124	miR-9 is involved in neural development, miR-132 regulates synaptic plasticity, and miR-124 is essential for neuronal differentiation.	Hébert, S. S., Horré, K., Nicolaï, L., Papadopoulou, A. S., Mandemakers, W., Silahtaroglu, A. N., .. & De Strooper, B. (2008). Loss of microRNA cluster miR-29a/b-1 in sporadic Alzheimer’s disease correlates with increased BACE1/β-secretase expression. *Proceedings of the National Academy of Sciences, 105*(17), 6415–6420. https://doi.org/10.1073/pnas.0710263105
				Lau, P., & de Strooper, B. (2013). Dysregulated microRNAs in neurodegenerative disorders. *Seminars in Cell & Developmental Biology, 21*(7), 768–773. https://doi.org/10.1016/j.semcdb.2010.01.009
				Gascon, E., & Gao, F. B. (2014). Cause or effect: Misregulation of microRNA pathways in neurodegeneration. *Frontiers in Neuroscience, 8*, 5. https://doi.org/10.3389/fnins.2014.00005
Metabolic disorders	Diabetes, obesity, and insulin resistance	miR-375, miR-143, miR-103	miR-375 regulates insulin secretion, miR-143 is involved in adipocyte differentiation, and miR-103 is associated with insulin sensitivity and glucose metabolism.	Poy, M. N., Eliasson, L., Krutzfeldt, J., Kuwajima, S., Ma, X., Macdonald, P. E., .. & Stoffel, M. (2004). A pancreatic islet-specific microRNA regulates insulin secretion. *Nature, 432*(7014), 226–230. https://doi.org/10.1038/nature03076
				Esau, C., Kang, X., Peralta, E., Hanson, E., Marcusson, E. G., Ravichandran, L. V., .. & Lollo, B. (2004). MicroRNA-143 regulates adipocyte differentiation. *Journal of Biological Chemistry, 279*(50), 52361–52365. https://doi.org/10.1074/jbc.C400438200
				Trajkovski, M., Hausser, J., Soutschek, J., Bhat, B., Akin, A., Zavolan, M., .. & Stoffel, M. (2011). MicroRNAs 103 and 107 regulate insulin sensitivity. *Nature, 474*(7353), 649–653. https://doi.org/10.1038/nature10112
Infectious diseases	Host-pathogen interactions and immune response modulation	miR-146a, miR-155, miR-122	miR-146a regulates immune responses to bacterial infections, miR-155 is involved in viral and bacterial infections, and miR-122 is essential for Hepatitis C virus replication.	O’Connell, R. M., Taganov, K. D., Boldin, M. P., Cheng, G., & Baltimore, D. (2007). MicroRNA-155 is induced during the macrophage inflammatory response. *Proceedings of the National Academy of Sciences, 104*(5), 1604–1609. https://doi.org/10.1073/pnas.0610731104
				Jopling, C. L., Yi, M., Lancaster, A. M., Lemon, S. M., & Sarnow, P. (2005). Modulation of hepatitis C virus RNA abundance by a liver-specific microRNA. *Science, 309*(5740), 1577–1581. https://doi.org/10.1126/science.1113329
				Lindsay, M. A. (2008). microRNAs and the immune response. *Trends in Immunology, 29*(7), 343–351. https://doi.org/10.1016/j.it.2008.04.004
Immune disorders	Biomarkers and therapeutic targets for lupus, rheumatoid arthritis, and MS	miR-146a, miR-155, miR-326	miR-146a is dysregulated in lupus and rheumatoid arthritis, miR-155 is involved in autoimmune inflammation, and miR-326 is associated with multiple sclerosis.	Pauley, K. M., Satoh, M., Chan, A. L., Bubb, M. R., Reeves, W. H., & Chan, E. K. (2008). Upregulated miR-146a expression in peripheral blood mononuclear cells from rheumatoid arthritis patients. *Arthritis Research & Therapy, 10*(4), R101. https://doi.org/10.1186/ar2493
				Thai, T. H., Calado, D. P., Casola, S., Ansel, K. M., Xiao, C., Xue, Y., .. & Rajewsky, K. (2007). Regulation of the germinal center response by microRNA-155. *Science, 316*(5824), 604–608. https://doi.org/10.1126/science.1141229
				Du, C., Liu, C., Kang, J., Zhao, G., Ye, Z., Huang, S., .. & Pei, G. (2009). MicroRNA miR-326 regulates TH-17 differentiation and is associated with the pathogenesis of multiple sclerosis. *Nature Immunology, 10*(12), 1252–1259. https://doi.org/10.1038/ni.1798
Regenerative medicine	Regulation of stem cell differentiation and pluripotency	miR-302, miR-145, miR-9	miR-302 maintains pluripotency in embryonic stem cells, miR-145 promotes differentiation, and miR-9 is involved in neural stem cell differentiation.	Suh, M. R., Lee, Y., Kim, J. Y., Kim, S. K., Moon, S. H., Lee, J. Y., .. & Kim, V. N. (2004). Human embryonic stem cells express a unique set of microRNAs. *Developmental Biology, 270*(2), 488–498. https://doi.org/10.1016/j.ydbio.2004.02.019
				Xu, N., Papagiannakopoulos, T., Pan, G., Thomson, J. A., & Kosik, K. S. (2009). MicroRNA-145 regulates OCT4, SOX2, and KLF4 and represses pluripotency in human embryonic stem cells. *Cell, 137*(4), 647–658. https://doi.org/10.1016/j.cell.2009.02.038
				Coolen, M., Katz, S., & Bally-Cuif, L. (2013). miR-9: A versatile regulator of neurogenesis. *Frontiers in Cellular Neuroscience, 7*, 220. https://doi.org/10.3389/fncel.2013.00220
Dermatology	Skin health, wound healing, and dermatological diseases	miR-21, miR-31, miR-132	miR-21 promotes fibroblast migration and wound closure, miR-31 regulates keratinocyte proliferation, and miR-132 enhances angiogenesis during wound healing.	Wang, T., Feng, Y., Sun, H., Zhang, L., Hao, L., & Shi, C. (2013). miR-21 regulates skin wound healing by targeting multiple aspects of the healing process. *American Journal of Pathology, 183*(6), 1931–1941. https://doi.org/10.1016/j.ajpath.2013.08.004
				Pastar, I., Khan, A. A., Stojadinovic, O., Lebrun, E., Medina, M. C., Brem, H., .. & Tomic-Canic, M. (2014). Induction of specific microRNAs inhibits cutaneous wound healing. *Journal of Biological Chemistry, 289*(35), 29324–29335. https://doi.org/10.1074/jbc.M114.564625
				Ban, E., Chung, S., Lee, M., Im, S., & An, S. (2018). MicroRNA-132 regulates angiogenesis in endothelial cells. *Journal of Biological Chemistry, 293*(1), 505–515. https://doi.org/10.1074/jbc.M117.799320
Pulmonary diseases	Biomarkers and therapeutic targets for asthma, COPD, and pulmonary fibrosis	miR-21, miR-155, miR-146a	miR-21 is associated with lung inflammation, miR-155 regulates immune responses in asthma, and miR-146a modulates fibrosis in pulmonary diseases.	Liu, G., Friggeri, A., Yang, Y., Milosevic, J., Ding, Q., Thannickal, V. J., & Kaminski, N. (2010). miR-21 mediates fibrogenic activation of pulmonary fibroblasts and lung fibrosis. *Journal of Experimental Medicine, 207*(8), 1589–1597. https://doi.org/10.1084/jem.20100035
				Pottelberge, G. R., Mestdagh, P., Bracke, K. R., Thas, O., van Pottelberge, G. R., van Thienen, R., .. & Joos, G. F. (2011). MicroRNA expression in induced sputum of smokers and patients with chronic obstructive pulmonary disease. *American Journal of Respiratory and Critical Care Medicine, 183*(7), 898–906. https://doi.org/10.1164/rccm.201002-0303OC
				Oglesby, I. K., Bray, I. M., Chotirmall, S. H., Stallings, R. L., O’Neill, S. J., McElvaney, N. G., & Greene, C. M. (2013). miR-126 is downregulated in cystic fibrosis airway epithelial cells and regulates TOM1 expression. *Journal of Immunology, 190*(7), 3898–3908. https://doi.org/10.4049/jimmunol.1202680
Gastroenterology	Biomarkers for inflammatory bowel disease (IBD) and gastrointestinal cancers	miR-21, miR-155, miR-146a	miR-21 is overexpressed in colorectal cancer, miR-155 is linked to IBD, and miR-146a regulates inflammation in gastrointestinal disorders.	Wu, W., He, X., Yang, L., Wang, Q., Li, X., & Zhang, Y. (2008). miRNA expression profiling of formalin-fixed paraffin-embedded (FFPE) colorectal cancer tissues. *International Journal of Clinical and Experimental Pathology, 1*(5), 400–406.
				Fasseu, M., Tréton, X., Guichard, C., Pedruzzi, E., Cazals-Hatem, D., Richard, C., .. & Ogier-Denis, E. (2010). Identification of restricted subsets of mature microRNA abnormally expressed in inactive colonic mucosa of patients with inflammatory bowel disease. *PLOS ONE, 5*(10), e13160. https://doi.org/10.1371/journal.pone.0013160
				Pekow, J., Dougherty, U., Mustafi, R., Zhu, H., Kocherginsky, M., Rubin, D. T., .. & Bissonnette, M. (2012). miR-155 is regulated by TGF-β1 in colonic epithelial cells and mediates TGF-β-induced down-regulation of interleukin-6. *American Journal of Physiology-Gastrointestinal and Liver Physiology, 302*(7), G1393–G1401. https://doi.org/10.1152/ajpgi.00426.2011
Oncofertility	Fertility preservation in cancer patients	miR-372, miR-515, miR-21	miR-372 is involved in spermatogenesis, miR-515 regulates trophoblast invasion, and miR-21 is associated with preeclampsia and fertility issues.	Morales-Prieto, D. M., Chaiwangyen, W., Ospina-Prieto, S., Schneider, U., Herrmann, J., Gruhn, B., & Markert, U. R. (2012). MicroRNA expression profiles of trophoblastic cells. *Placenta, 33*(9), 725–734. https://doi.org/10.1016/j.placenta.2012.06.012
				Lv, Y., Lu, C., Ji, X., Miao, Z., Long, W., Ding, H., & Tang, J. (2018). Roles of microRNAs in preeclampsia. *Journal of Cellular Physiology, 234*(2), 1052–1061. https://doi.org/10.1002/jcp.27091
				Zhang, Y., Diao, Z., Su, L., Sun, H., Li, R., Cui, H., .. & Hu, Y. (2019). MicroRNA-155 contributes to preeclampsia by down-regulating CYR61. *American Journal of Obstetrics and Gynecology, 220*(5), 497.e1–497.e12. https://doi.org/10.1016/j.ajog.2019.01.004
Endocrinology	Regulation of hormone-related disorders (e.g., diabetes, thyroid disorders)	miR-375, miR-143, miR-103	miR-375 regulates insulin secretion, miR-143 is involved in adipocyte differentiation, and miR-103 is associated with insulin sensitivity.	Poy, M. N., Spranger, M., Stoffel, M. (2004). microRNAs: tiny regulators with great potential. *Cell, 122*(6), 837–840. https://doi.org/10.1016/j.cell.2005.02.034
				Esau, C., Davis, S., Yu, X. X., Pandey, S. K., Pear, M., & Monia, B. P. (2006). miR-122 regulation of lipid metabolism revealed by in vivo antisense targeting. *Cell Metabolism, 3*(2), 87–98. https://doi.org/10.1016/j.cmet.2006.01.005
				Trajkovski, M., Hausser, J., Soutschek, J., Bhat, B., Zavolan, M., & Heim, M. H. (2011). MicroRNAs 103 and 107 regulate insulin sensitivity. *Nature, 474*(7353), 649–653. https://doi.org/10.1038/nature10112
Orthopedics	Bone health, fracture healing, and osteoarthritis	miR-21, miR-140, miR-146a	miR-21 promotes osteoblast differentiation, miR-140 is involved in cartilage homeostasis, and miR-146a regulates inflammation in osteoarthritis.	Li, H., Xie, H., Liu, W., Hu, R., Huang, B., Tan, Y. F., .. & Wu, X. P. (2009). A novel microRNA targeting HDAC5 regulates osteoblast differentiation in mice and contributes to primary osteoporosis in humans. *Journal of Clinical Investigation, 119*(12), 3666–3677. https://doi.org/10.1172/JCI39329
				Miyaki, S., Sato, T., Inoue, A., Otsuki, S., Ito, Y., Yokoyama, S., .. & Asahara, H. (2010). MicroRNA-140 plays dual roles in both cartilage development and homeostasis. *Genes & Development, 24*(11), 1173–1185. https://doi.org/10.1101/gad.1915510
				Yamasaki, K., Nakasa, T., Miyaki, S., Ishikawa, M., Deie, M., Adachi, N., .. & Ochi, M. (2009). Expression of MicroRNA-146a in osteoarthritis cartilage. *Arthritis & Rheumatism, 60*(4), 1035–1041. https://doi.org/10.1002/art.24404
Ophthalmology	Biomarkers for eye diseases (e.g., glaucoma, macular degeneration)	miR-146a, miR-155, miR-124	miR-146a regulates inflammation in retinal diseases, miR-155 is involved in ocular immune responses, and miR-124 is essential for retinal development.	Lukiw, W. J., & Pogue, A. I. (2007). Induction of specific micro RNA (miRNA) species by ROS-generating metals implicated in aging and age-related diseases. *International Journal of Molecular Sciences, 8*(5), 923–940. https://doi.org/10.3390/ijms8050923
				Wei, Y., Nazari-Jahantigh, M., Neth, P., Weber, C., & Schober, A. (2013). MicroRNA-126, − 145, and −155: a therapeutic triad in atherosclerosis? *Arteriosclerosis, Thrombosis, and Vascular Biology, 33*(3), 449–454. https://doi.org/10.1161/ATVBAHA.112.300432
				Karali, M., Peluso, I., Gennarino, V. A., Bilio, M., Verde, R., Lago, G., .. & Banfi, S. (2010). miRNeye: a microRNA expression atlas of the mouse eye. *BMC Genomics, 11*, 715. https://doi.org/10.1186/1471-2164-11-715
Cancer immunotherapy	Enhancing immune response against cancer	miR-34a, miR-155, miR-21	miR-34a enhances T-cell activity, miR-155 regulates immune cell function, and miR-21 inhibition improves immunotherapy outcomes.	Janssen, H. L., Reesink, H. W., Lawitz, E. J., Zeuzem, S., Rodriguez-Torres, M., Patel, K., .. & Hodges, M. R. (2013). Treatment of HCV infection by targeting microRNA. *New England Journal of Medicine, 368*(18), 1685–1694. https://doi.org/10.1056/NEJMoa1209026
				Bouchie, A. (2013). First microRNA mimic enters clinic. *Nature Biotechnology, 31*(7), 577. https://doi.org/10.1038/nbt0713-577
				Rupaimoole, R., & Slack, F. J. (2017). MicroRNA therapeutics: Towards a new era for the management of cancer and other diseases. *Nature Reviews Drug Discovery, 16*(3), 203–222. https://doi.org/10.1038/nrd.2016.246
Pharmacogenomics	Personalized medicine and drug response prediction	miR-21, miR-155, miR-34a	miR-21 is associated with drug resistance, miR-155 regulates drug metabolism, and miR-34a is used in miRNA-based therapeutics.	Rukov, J. L., & Shomron, N. (2014). MicroRNA pharmacogenomics: Post-transcriptional regulation of drug response. *Trends in Molecular Medicine, 17*(8), 412–423. https://doi.org/10.1016/j.molmed.2011.04.001
				van Rooij, E., & Olson, E. N. (2012). MicroRNA therapeutics for cardiovascular disease: Opportunities and obstacles. *Nature Reviews Drug Discovery, 11*(11), 860–872. https://doi.org/10.1038/nrd3864
				Rupaimoole, R., & Slack, F. J. (2017). MicroRNA therapeutics: Towards a new era for the management of cancer and other diseases. *Nature Reviews Drug Discovery, 16*(3), 203–222. https://doi.org/10.1038/nrd.2016.246
Aging	Biomarkers and modulators of aging processes	miR-34a, miR-146a, miR-21	miR-34a is involved in cellular senescence, miR-146a regulates inflammation during aging, and miR-21 is associated with age-related diseases.	Bhaumik, D., Scott, G. K., Schokrpur, S., Patil, C. K., Orjalo, A. V., Rodier, F., .. & Campisi, J. (2009). MicroRNAs miR-146a/b and miR-21 regulate the senescence-associated inflammatory response. *Nature Cell Biology, 11*(9), 1154–1161. https://doi.org/10.1038/ncb1965
				Olivieri, F., Rippo, M. R., Procopio, A. D., & Fazioli, R. (2012). Circulating inflamma-miRs in aging and age-related diseases. *Frontiers in Genetics, 3*, 320. https://doi.org/10.3389/fgene.2012.00320
				Smith-Vikos, T., & Slack, F. J. (2012). MicroRNAs and their roles in aging. *Journal of Cell Science, 125*(1), 7–17. https://doi.org/10.1242/jcs.099200

Summarizes the roles of miRNAs in various diseases, highlighting their functions in diagnostics, prognosis, and treatment. It showcases key miRNAs linked to specific conditions and their biological impact, emphasizing their potential in biomarker discovery and precision medicine.

## Imaging-based approaches

Imaging-based approaches, particularly those utilizing ML algorithms, have shown tremendous promise in predicting metastasis across various cancer types. These technologies have the potential to enhance diagnostic accuracy, allowing clinicians to detect metastatic spread earlier and more reliably. One of the most exciting applications of ML in imaging is magnetic resonance imaging (MRI)-based models, which have demonstrated a strong capacity to stratify patients based on tumor characteristics and metastatic risk. For instance, in oral tongue squamous cell carcinoma (OTSCC), MRI-based models driven by ML algorithms have exhibited the ability to classify patients according to the pT stage with a high degree of accuracy^[^22^]^. These models also show promise in predicting cervical lymph node metastasis (CLNM), an important factor in determining treatment strategies and prognosis^[^23-25^]^. The use of shape-based and intensity-based features extracted from MRI images has the potential to improve the sensitivity of models in predicting CLNM, as these features can capture key morphological and textural differences between metastatic and non-metastatic tissues.

However, despite these advances, the current level of accuracy for MRI-based models in predicting metastasis remains suboptimal. One challenge is the complexity of tumor biology, which often cannot be fully captured through shape- or intensity-based features alone. To address this, researchers are refining imaging models by incorporating more sophisticated approaches, such as MRI-based radiomics. Radiomics involves the extraction of a large number of quantitative features from medical images, providing a more detailed analysis of tumor heterogeneity and behavior^[^23^]^. By integrating radiomic features with ML algorithms, researchers hope to improve the predictive power of these models, making them more reliable in clinical settings. Additionally, the introduction of other predictors, such as genomic data or patient-specific clinical information, could further enhance the accuracy and applicability of MRI-based models for metastasis prediction.

Recent advancements in quantitative cellular imaging have also contributed to the ability to detect metastasis-related changes at the cellular level. Quantitative cellular imaging allows for the detection of specific morphological phenotype changes that are indicative of a metastatic cell state. These phenotype changes are connected to underlying complex signaling pathways, which drive cancer progression and metastasis. ML and deep learning techniques have been employed to analyze these morphological features, offering new insights into the metastatic potential of cells. For example, cellular morphology – such as shape, size and texture – can provide a robust readout of metastatic potential, as these traits are often altered in cancer cells undergoing metastasis^[^24^]^.

In one notable study, researchers used two-dimensional cell culture to demonstrate that cell shape can serve as a reliable indicator of metastatic cell state. By analyzing cell shape in real time, researchers were able to predict whether cancer cells were more likely to metastasize^[^25-27^]^. This kind of phenotypic profiling is particularly useful for identifying aggressive tumors at an early stage. Another study advanced this concept further by developing an autoencoder using a deep convolutional neural network (CNN) to encode latent cell information from imaging data^[^25^]^. This deep learning model successfully discriminated between high and low metastatic melanoma patient-derived xenografts based on their cellular characteristics. The use of CNNs, which are particularly adept at identifying complex patterns in imaging data, represents a significant leap forward in imaging-based metastasis prediction.

Deep learning algorithms, including CNNs and other advanced techniques like autoencoders, have become integral to the analysis of large and complex datasets in cancer imaging^[^25^]^. These algorithms can process vast amounts of data from medical images and cellular imaging platforms, identifying subtle features that are imperceptible to human observers. In the context of metastasis, this can include identifying slight variations in tumor texture, microenvironmental changes or alterations in vascularization, all of which could signal the likelihood of metastasis. Additionally, deep learning algorithms can be trained to integrate data from multiple imaging modalities – such as MRI, computed tomography (CT) and positron emission tomography (PET) – to provide a more comprehensive assessment of tumor characteristics and metastatic risk^[^26^]^.

One of the most promising aspects of imaging-based metastasis prediction is the ability to combine these advanced imaging techniques with other data sources, such as molecular and genomic biomarkers. Multimodal models that integrate imaging data with molecular profiling are likely to offer the most accurate predictions, as they provide a more holistic view of the tumor and its metastatic potential. For example, combining imaging features with data on gene expression, mutation profiles and protein biomarkers could allow clinicians to not only predict whether a tumor is likely to metastasize but also understand the specific biological mechanisms driving the metastasis^[^27^]^. This integrated approach is a critical step toward personalized medicine, where treatments are tailored to the individual patient’s unique tumor profile.

Furthermore, the application of AI in imaging-based metastasis prediction has spurred the development of explainable AI (XAI) techniques, which aim to make complex models more interpretable for clinicians. While deep learning models, such as CNNs, are highly effective at pattern recognition, they often operate as “black boxes,” meaning that their decision-making processes are not easily understood. XAI techniques aim to provide transparency, allowing clinicians to understand why a model made a particular prediction^[^28^]^. This is particularly important in a clinical setting, where decisions about metastasis can have significant implications for treatment and prognosis.

In the context of metastasis, XAI can help explain which features of an image contributed most to the model’s prediction, whether those features are related to tumor size, shape, intensity or cellular morphology. By making AI models more interpretable, XAI can increase clinicians’ confidence in using these models as decision-support tools, ultimately improving patient outcomes. The future of imaging-based metastasis prediction lies not only in improving the accuracy of models but also in making them more user-friendly and interpretable for healthcare professionals^[^29^]^.

Finally, imaging-based approaches are also shedding light on the underlying mechanisms of cancer progression and metastasis. By analyzing how tumors evolve over time and how cancer cells interact with their microenvironment, imaging models are helping to uncover the key drivers of metastasis. For example, changes in the tumor’s vascularization, immune cell infiltration and stromal interactions can all be visualized through advanced imaging techniques, providing new insights into the processes that enable cancer cells to spread to distant organs^[^30^]^. These insights are critical for developing new therapeutic strategies that target the metastatic process, such as inhibiting angiogenesis or modulating the tumor microenvironment.

## Traditional statistical models in predictive oncology

The foundation of predictive analytics in cancer has long been traditional statistical models, which provide interpretable frameworks for correlating clinical, genetic, and demographic factors with outcomes like treatment response, recurrence, and survival. Among the most used techniques, the Cox proportional hazards model has proven essential to survival analysis since it enables researchers to evaluate risk factors while taking censored data into consideration^[^30^]^. For instance, in the landmark TAILORx trial, Sparano *et al* used Cox regression to stratify patients with early-stage breast cancer by recurrence risk using the 21-gene Oncotype DX assay, demonstrating that age and hormone status significantly impacted survival outcomes. However, the model’s applicability in situations with time-dependent covariates, like changing tumor mutational burden during immunotherapy, is limited by its dependence on the proportional hazards assumption (Rizvi *et al*).

Logistic regression, another essential method, is frequently used to predict binary outcomes like chemotherapy resistance or remission. KRAS mutations were found to be predictive of platinum-based chemotherapy resistance in non-small cell lung cancer (NSCLC) by Skoulidis *et al* using logistic regression, with an odds ratio of 2.1 (*P* < 0.001). Despite its simplicity and clinical interpretability, logistic regression assumes linear relationships between predictors and outcomes, which may oversimplify complex biological interactions (Tibshirani, 1996). Non-parametric approaches like the Kaplan–Meier estimator circumvent distributional assumptions and are widely used to visualize survival differences between subgroups. Hodi *et al* compared the progression-free survival (PFS) of patients with advanced melanoma treated with nivolumab–ipilimumab combination therapy to monotherapy in the CheckMate 067 trial using Kaplan–Meier curves (*P* < 0.001)^[^30^]^. While intuitive, Kaplan–Meier analyses are unable to adjust for confounding variables, rendering them inadequate for multivariate precision oncology (Clark *et al*).

Techniques for penalized regression, including LASSO (Least Absolute Shrinkage and Selection Operator), have become popular in response to the difficulties presented by high-dimensional omics evidence. Using LASSO, Waldron *et al* improved a 200-gene predictive signature for ovarian cancer, obtaining an external validation concordance index of 0.71. Despite reducing overfitting, penalized approaches impose subjectivity and computational complexity due to their dependence on hyperparameter adjustment (e.g., λ in LASSO) (Friedman *et al*).

Despite their limitations, traditional models remain indispensable for hypothesis-driven research and regulatory decision-making^[^31^]^. For example, Cox regression underpinned the FDA approval of pembrolizumab for MSI-H/dMMR cancers, as demonstrated by Le *et al*. Recent advancements, such as time-dependent Cox models (Austin *et al*) and hybrid machine learning-statistical frameworks (Christodoulou *et al*), aim to address traditional weaknesses while preserving interpretability^[^31^]^. However, the trade-off between simplicity and biological realism persists, particularly in the era of multi-omics integration. The comparison of traditional statistical models is provided in (Table 3).

**Table 3:** Applications, strengths, and limitations of traditional statistical models in oncology^[^43^-^52,83^-^89^]^

Model	Applications	Strengths	Limitations	Examples
Cox proportional hazards	Survival analysis, risk stratification, treatment effect estimation.	Adjusts for covariates; handles censored data.	Assumes proportional hazards; struggles with time-varying effects.	Sparano *et al*, 2018 (NEJM): Oncotype DX in breast cancer.
Logistic regression	Binary outcome prediction (e.g., recurrence, resistance).	Provides odds ratios; easy to interpret.	Assumes linearity; prone to overfitting.	Skoulidis *et al*, 2015 (NEJM): KRAS mutations in NSCLC.
Kaplan–Meier estimator	Non-parametric survival curves for subgroup comparisons.	No distributional assumptions; visual clarity.	Ignores covariates; limited to univariate analysis.	Hodi *et al*, 2016 (NEJM): CheckMate 067 trial in melanoma.
Penalized regression (LASSO)	Feature selection in high-dimensional omics data.	Reduces overfitting; handles multicollinearity.	Requires hyperparameter tuning; less intuitive.	Waldron *et al*, 2011 (JCO): Ovarian cancer gene signatures.

## Machine learning approaches and supervised learning algorithms

ML has emerged as a transformative tool in predictive modeling for cancer metastasis, offering the ability to process and analyze vast amounts of complex data to make accurate predictions about cancer progression and metastatic potential. By leveraging a variety of sophisticated algorithms, ML enables the identification of patterns within clinical, genomic, imaging and molecular datasets that may not be readily apparent to human clinicians^[^30^]^. This advanced analysis provides critical insights into cancer behavior, facilitating earlier detection of metastasis, better treatment planning and more personalized care for patients. As researchers continue to refine these techniques, ML is poised to play an increasingly central role in the fight against metastatic cancer.

Among the most widely used ML approaches in metastasis prediction are supervised learning algorithms, which are trained on labeled data to learn the relationships between input features and specific outcomes, such as whether a tumor is likely to metastasize. These algorithms have demonstrated high levels of accuracy in predicting metastasis, often outperforming traditional statistical models due to their ability to capture complex interactions between variables. Several supervised learning methods have shown particular promise in metastasis prediction, including advanced techniques like Light Gradient Boosting Machine (LightGBM), Categorical Boosting (CatBoost), Extreme Gradient Boosting (XGBoost), Gradient Boosting Trees (GBT) and AdaBoost. These boosting algorithms work by combining the outputs of multiple weak classifiers to create a stronger predictive model^[^31^]^. Each successive model is trained to correct the errors made by the previous one, resulting in an ensemble model with enhanced accuracy. These algorithms are particularly effective in handling high-dimensional data, making them ideal for analyzing large cancer datasets that contain numerous clinical, pathological and genomic features.

Random Forest (RF) is one of the most popular ensemble learning techniques used in predictive modeling for cancer metastasis. It constructs multiple decision trees by bootstrapping from the training dataset, meaning that it selects random subsets of features and samples to build each tree. The final prediction is made by aggregating the predictions from all the trees, which helps to reduce overfitting and improve generalization. The Gini index is typically used as the splitting criterion in RF, helping to determine the most important features for classification^[^32^]^. Gini importance, also known as Mean Decrease Impurity (MDI), is used to rank the importance of different features in predicting metastasis. This technique is particularly useful for understanding which clinical or genomic factors are most influential in driving cancer spread, allowing researchers and clinicians to focus on key variables that impact patient outcomes.

Support vector machines (SVMs) are another widely used technique in cancer metastasis prediction. SVMs work by mapping input features into a higher-dimensional space and identifying a hyperplane that maximizes the margin between classes, such as metastatic and non-metastatic tumors. This approach is particularly effective in handling data with multiple variables and has shown success in predicting metastasis in various cancers. By effectively separating different classes based on the input features, SVMs can predict which tumors have a higher likelihood of metastasizing^[^33^]^. Additionally, they are useful in combination with other ML techniques for improving accuracy in complex, multi-feature datasets.

Another machine learning approach showing promise in this area is neural networks (NNs). Deep learning architectures, such as convolutional neural networks (CNNs) and recurrent neural networks (RNNs), have been extensively applied in cancer research, particularly in predicting drug synergy and treatment outcomes^[^34^]^. These networks have the capacity to model non-linear relationships and learn intricate patterns in large datasets, making them ideal for predicting cancer metastasis. For example, CNNs have been particularly effective when applied to imaging datasets, identifying subtle features in tumor morphology that correlate with metastatic potential. RNNs, on the other hand, are effective in analyzing sequential data, making them useful for tracking the progression of metastatic disease over time.

Logistic regression, although simpler than other algorithms like neural networks, remains an effective tool in the predictive modeling of cancer metastasis. It has been particularly useful in determining the highest contributing variables for patient adherence to treatment and assessing the likelihood of metastasis based on these variables^[^35^]^. While its predictive power is more limited compared to more complex models, logistic regression’s simplicity and interpretability make it a valuable tool in clinical settings, where understanding the contributing factors behind predictions is crucial.

Naive Bayes is another algorithm frequently used in cancer metastasis prediction due to its probabilistic nature. Based on Bayes’ theorem, Naive Bayes is known for its fast and robust performance, particularly when dealing with large, high-dimensional datasets common in cancer research. It operates by calculating the probability of each class (e.g., metastatic or non-metastatic) based on the distribution of input features, offering a reliable and efficient means of classifying tumors. Its simplicity and computational efficiency make it a popular choice for preliminary analyses or when working with limited computational resources^[^36^]^.

Together, these ML algorithms provide a powerful suite of tools for predicting cancer metastasis. Whether through the use of boosting algorithms, neural networks or simpler models like logistic regression and Naive Bayes, ML approaches are revolutionizing the ability to forecast cancer progression. As these technologies continue to advance, they will likely become increasingly integrated into clinical practice, aiding oncologists in making more informed, data-driven decisions to improve patient outcomes.

## Feature selection methods and model performance metrics

Feature selection plays a pivotal role in enhancing the robustness of ML models and mitigating the risk of overfitting, particularly in predictive modeling for metastasis in oncology. Various feature selection techniques are commonly employed to identify the most relevant variables while reducing the dimensionality of the dataset. One such method is the Least Absolute Shrinkage and Selection Operator (LASSO), widely regarded for its strong ability to reduce covariates by imposing penalties on the absolute size of regression coefficients. Additionally, the Intraclass Correlation (ICC) and Concordance Correlation Coefficient (CCC) are often applied in the initial stages of model development to assess the reproducibility of features, ensuring that the selected features are both reliable and consistent^[^37^]^. Elastic Net, a hybrid of L1 and L2 regularization techniques, is another popular method, combining the benefits of both regularization forms to perform feature selection and coefficient narrowing^[^38^]^. This is particularly advantageous when working with datasets that contain numerous related features, as it balances sparsity and group selection effectively.

In predictive modeling using medical imaging data, radiomic feature extraction is crucial. Deep learning models, particularly CNNs, can extract complex radiomic features from CT and MRI images. These features capture important patterns within tumor structures, which can then be used to predict metastasis with greater accuracy. Often, feature selection can be performed using single, serial or parallel approaches, and in some studies, as many as 13 feature selection methods have been employed in parallel to ensure the most relevant variables are chosen for prediction^[^37,38^]^. This comprehensive approach maximizes the model’s ability to detect meaningful patterns and reduces noise, thus improving overall model performance^[^38^]^.

Once features have been selected, it is essential to assess the predictive abilities of ML models using various performance evaluation metrics. Accuracy, one of the most commonly used metrics, measures the overall correctness of a model’s predictions, but it may not always be sufficient, especially in imbalanced datasets. For this reason, the F1 score, which is the harmonic mean of precision and recall, offers a more balanced assessment^[^38^]^. Precision measures the proportion of true positive predictions relative to all positive predictions, while recall (sensitivity) calculates the ratio of true positives to all actual positive instances. In addition, the Area Under the ROC (Receiver Operating Curve) is a valuable metric for evaluating a model’s ability to distinguish between different classes, providing insights into its discriminative power. Another key metric is the Brier score, which evaluates the reliability of probabilistic predictions by comparing predicted probabilities to the actual outcomes^[^39^]^. A lower Brier score indicates a model with high predictive reliability, often preferred in clinical settings. Model performance is assessed using various metrics, as shown in Table 4.

**Table 4:** Performance metrics for predictive models^[^44^-^60,83^-^89^]^

Metric	Formula/definition	Clinical relevance	References
Accuracy	(TP + TN)/(TP + TN + FP + FN)	Overall correctness; limited utility in imbalanced data.	^[^72^]^
F1 score	2 × Precision × RecallPrecision + Recall2 × Precision + RecallPrecision × Recall	Balances false positives/negatives (critical for cancer prognosis).	^[^72,73^]^
AUC-ROC	Area under ROC curve	Discriminative power (0.5 = random, 1.0 = perfect).	^[^73,74^]^
Brier score	1 N∑i =1 N(pi−oi)2*N*1 ∑*i* =1 *N* (*pi* −*oi*)2	Reliability of probabilistic predictions (<0.25 = clinically reliable).	^[^75,76^]^

The table provides key evaluation metrics used to assess predictive models in cancer prognosis. It includes Accuracy, F1 Score, AUC-ROC, and Brier Score, with their respective formulas, clinical relevance, and supporting references.

In one study, the Light Gradient Boosting Machine (LightGBM) algorithm demonstrated remarkable efficacy, achieving an accuracy of 96% and an AUC value of 99.3%^[^40^]^. The Brier score for this model was 0.024, significantly below the generally accepted threshold of 0.25, highlighting its robustness and reliability in predicting metastasis^[^41^]^. However, beyond the metrics of the model’s performance on training datasets, it is essential to ensure generalizability. To achieve this, model validation followed by independent testing using external datasets is crucial. This process evaluates the model’s performance on new, unseen data, providing a more realistic estimate of its predictive capabilities and ensuring that the model is not overfitting to the training data^[^42^]^. Robust validation procedures, combined with rigorous feature selection and evaluation, ensure that machine learning models can effectively predict cancer metastasis and contribute to improved clinical decision-making. Feature selection methods play a key role in refining predictive models (Fig. 4).

## Radiomics and imaging features

Radiomics has become a rapidly expanding field, capturing significant interest within the research and clinical communities due to its potential to predict treatment outcomes and unravel cancer genetics through non-invasive means. This advanced approach allows researchers and clinicians to extract quantitative features from readily available radiological images such as CT, MRI and PET scans. These features, which include parameters like intensity, shape, volume and texture, provide a deeper insight into tumor phenotypes than traditional imaging techniques^[^43^]^. The ability to capture and quantify these aspects offers a more granular characterization of the tumor microenvironment, making radiomics a promising tool for personalized cancer care.

One of the key advantages of radiomics is its ability to translate medical images into high-dimensional data that can be mined for patterns and correlations. For instance, the shape of a tumor, its surface irregularities or even the distribution of intensity within a radiological scan can provide critical insights into the underlying biological processes, such as tumor growth, angiogenesis or cellular architecture. These image-derived features can reflect complex biological information, including tumor cell morphology, gene expression profiles and the degree of tumor heterogeneity. This is especially valuable in the field of oncology, where heterogeneity within the tumor microenvironment plays a crucial role in treatment response and disease progression^[^44^]^. Radiomics provides a way to quantify and visualize this heterogeneity, which is often invisible to the human eye, thereby aiding in more accurate prognosis and treatment planning.

Recent studies have demonstrated the utility of radiomics-based biomarkers in predicting not only cancer progression but also treatment responses at the lesion level. For instance, in cases of liver metastases, radiomic features extracted from CT images have shown strong correlations with pathological features, offering a non-invasive alternative for assessing tumor biology^[^45^]^. This is especially relevant in patients undergoing systemic treatments such as chemotherapy or immunotherapy, where predicting lesion-level treatment response is critical for tailoring therapies. In the realm of immunotherapy, where response rates can be highly variable, lesion-level prediction using radiomic features has been explored across multiple metastatic sites, with encouraging results^[^46^]^. These findings suggest that radiomics could serve as a valuable tool in refining treatment strategies, allowing for a more personalized approach based on individual lesion characteristics rather than a one-size-fits-all methodology.

Radiomics not only predicts treatment response but also holds promise for early detection of metastasis and even assessing the risk of recurrence. By detecting subtle variations in texture, shape, or intensity within a tumor, radiomic analysis can identify early signs of metastasis before they become clinically evident. This capability is particularly important in cancers where early intervention dramatically improves survival rates^[^47^]^. Moreover, radiomics has been explored for its role in predicting patient outcomes by assessing tumor heterogeneity. Tumors with greater heterogeneity tend to be more aggressive and resistant to therapy and quantifying this through radiomic features provides clinicians with a more detailed understanding of a tumor’s behavior.

The ability of radiomics to serve, as a non-invasive biomarker for cancer is further strengthened by its integration with ML and AI. ML algorithms can analyze the vast amount of radiomic data generated from medical images, identifying patterns and relationships that are too complex for traditional statistical methods^[^48^]^. For example, machine learning models have been employed to predict immunotherapy outcomes by correlating radiomic features with immune response markers. These AI-driven radiomics models can provide predictions about treatment efficacy, tumor recurrence and overall survival, helping clinicians make more informed decisions about patient care.

Additionally, radiomics has shown potential in correlating imaging features with genomic and molecular data, a concept known as radiogenomics. By linking radiomic features to gene expression profiles, radiogenomics offers a window into the molecular landscape of tumors without the need for invasive biopsies. This emerging field has demonstrated that certain radiomic features may be associated with specific genetic mutations or pathways involved in cancer progression^[^49^]^. For instance, studies have identified correlations between radiomic features and mutations in key cancer-driving genes like EGFR, KRAS and TP53. This integration of radiomic data with genetic information could eventually lead to more precise and personalized treatment strategies based on a patient’s specific tumor biology.

The applications of radiomics are broadening beyond just treatment prediction and genetic correlation. It is also being explored in clinical trial settings to evaluate novel therapies and to serve as a companion diagnostic tool. By providing an objective, quantitative assessment of treatment response over time, radiomics can help in identifying early responders and non-responders in clinical trials, streamlining the drug development process^[^50^]^. In radiation oncology, radiomics is being used to optimize treatment planning by predicting which areas of the tumor are most likely to be resistant to therapy, allowing for more targeted radiation doses.

## Clinical and molecular data fusion

The fusion of clinical and molecular data has emerged as a transformative approach in the field of oncology, demonstrating significant potential for enhancing predictive models of cancer metastasis. By integrating traditional clinical information – such as patient demographics, histopathological features, and treatment histories – with molecular data encompassing gene expression profiles, protein expression levels and DNA methylation patterns, researchers are able to construct more comprehensive and accurate predictive models^[^51^]^. This multidimensional approach provides a holistic view of the tumor microenvironment and allows for the identification of intricate relationships that may influence cancer progression and treatment response.

One of the key strategies employed in this integrated approach is feature-level fusion, where features from different omic levels are combined to create a unified dataset for classification modeling. In these models, features originating from various sources are concatenated to produce a fused feature representation. This method capitalizes on the complementary nature of the different types of data, enabling the model to leverage a broader spectrum of information. For instance, integrating gene expression data with imaging-derived radiomic features can provide insights into the biological behavior of tumors that might not be captured by either dataset alone. This comprehensive feature set can then be used to train sophisticated classification algorithms, ultimately improving the model’s predictive power and accuracy^[^52^]^.

In addition to feature-level fusion, decision-level fused classification models have gained traction in the pursuit of accurate cancer prediction. These models operate by training classifiers on each molecular level of evidence independently, while also incorporating features from combined levels of data. By employing this strategy, decision-level fusion effectively aggregates the strengths of individual classifiers, allowing for the synthesis of predictions that reflect the multifaceted nature of cancer biology. Studies have demonstrated that classifiers trained with feature-level fused molecular features often outperform those that rely solely on single-omic data^[^53^]^. This enhanced classification accuracy is crucial, as it can lead to better stratification of patients, more tailored treatment plans and ultimately improved patient outcomes.

The integration of multiple data types – including radiomics, multi-omics and clinical data – has shown particularly promising results in predictive modeling for cancer metastasis. Recent research has indicated that models utilizing this multi-faceted approach consistently demonstrate improved predictive performance compared to those relying on single data types. For instance, combining radiomic features from imaging studies with genomic and transcriptomic data can yield richer insights into the tumor’s molecular landscape, providing a more nuanced understanding of its behavior and potential response to therapies^[^54^]^. Such comprehensive models not only enhance predictive accuracy but also facilitate the identification of novel biomarkers that can be targeted for therapeutic intervention.

Moreover, the fusion of clinical and molecular data offers the potential to deepen our understanding of cancer metastasis itself. By correlating clinical outcomes with molecular characteristics, researchers can uncover patterns that reveal how specific genetic alterations or epigenetic modifications may drive metastatic spread. This knowledge is invaluable for developing targeted therapies and interventions tailored to the unique molecular profile of each patient’s tumor. Personalized medicine, informed by a thorough integration of data types, holds the promise of more effective treatments that can significantly improve patient survival rates and quality of life.

The application of advanced ML algorithms further enhances the potential of integrated data approaches. These algorithms excel in handling high-dimensional data and are adept at uncovering complex, non-linear relationships among features. Machine learning techniques can efficiently process the vast amounts of information generated from multi-omics and clinical datasets, enabling the identification of predictive signatures that may have otherwise gone unnoticed^[^55^]^. As a result, ML models trained on fused datasets are increasingly employed in clinical practice, helping clinicians make more informed decisions regarding treatment strategies and patient management.

**Figure 3.**
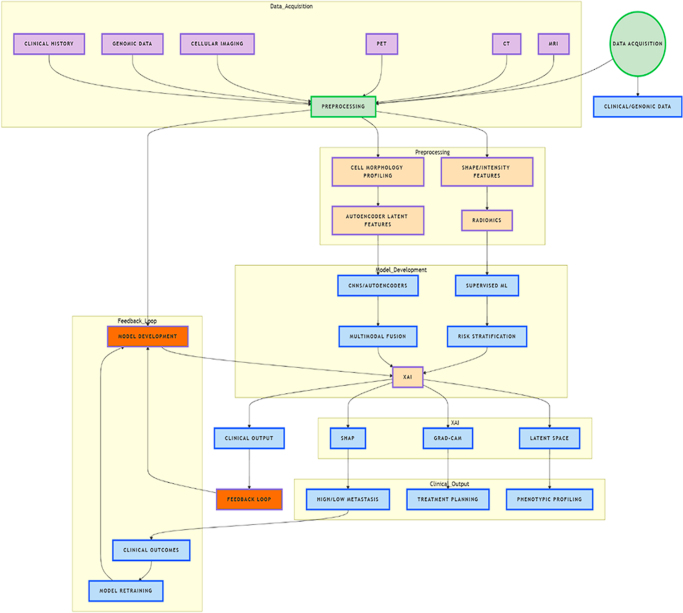
Prediction and clinical decision-making^[^49,50,83-85^]^. Diagram illustrates the integration of clinical, genomic, and imaging data with machine learning and XAI to enhance metastasis prediction and clinical decision-making.

In conclusion, the fusion of clinical and molecular data represents a significant advancement in the predictive modeling of cancer metastasis. By integrating various data types, including gene expression, proteomic profiles, radiomic features and traditional clinical information, researchers can develop models that offer a more comprehensive understanding of cancer biology. Feature-level and decision-level fusion techniques enhance predictive accuracy and contribute to the identification of novel biomarkers, while advanced machine learning algorithms enable the efficient processing of complex data. Ultimately, this integrated approach not only improves our understanding of cancer metastasis but also holds the potential to revolutionize patient care through personalized treatment strategies and improved clinical outcomes^[^56^]^. As the field continues to evolve, ongoing research into data fusion methodologies will be critical in driving further innovations in cancer diagnosis, treatment and prognostication. Multi-omics integration enhances predictive modeling (Fig. 3).

**Figure 4.**
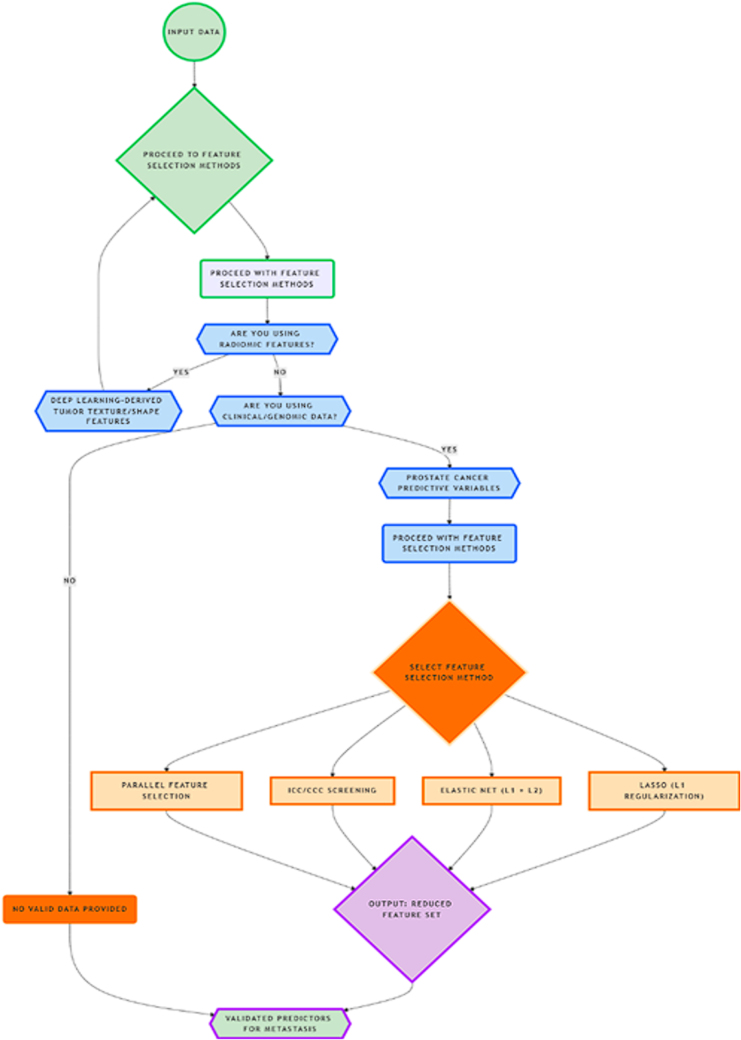
Feature selection workflow for metastasis prediction^[^55-61^]^. Flowchart outlines the feature selection process for metastasis prediction, integrating radiomic, genomic, and clinical data. It guides the selection of predictive variables, applying methods such as LASSO, Elastic Net, and ICC/CCC screening, ensuring an optimized and validated feature set.

## Prospective clinical trials and regulatory considerations

Prospective clinical trials are essential for validating predictive models and assessing their impact on patient outcomes, playing a critical role in the development of effective cancer therapies. These trials help researchers identify therapeutic signals, thereby providing a clearer understanding of the spectrum of clinically relevant toxicities associated with new treatments. In contemporary phase I trials, which often involve drugs developed with a profound understanding of biology and biomarker-based patient selection, one of the foremost objectives is to detect these therapeutic signals. To optimize the efficiency and efficacy of phase I trials, several innovative strategies have been implemented. These include using toxicity-adjusted dose escalation instead of traditional, predetermined Fibonacci-related schemas, allowing for increases in the number of patients at each dose level and implementing expansion cohorts^[^57^]^. Furthermore, telescoped trials that transition directly from phase I to phase II – and potentially to phase III – have emerged as promising methodologies. These approaches not only expedite drug development but also enhance the ability to gauge treatment efficacy while ensuring that safety standards are maintained.

Regulatory authorities play a pivotal role in overseeing the validation and implementation of predictive models within clinical practice. Key considerations for regulatory compliance include ensuring the technical robustness and safety of the models, as accuracy and reliability are paramount when outputs affect human subjects in clinical trials. Transparency in data usage is also critical, necessitating clear information about the representativeness of the data used to train algorithms and the intended applications of these models^[^58^]^. Moreover, traceability and data governance are essential, with stringent procedures required to meet relevant standards for data management throughout the trial lifecycle. Explainability is another crucial aspect, as regulatory bodies must receive clear statements regarding the model outputs and their intended use. Equally important is the need for human oversight, ensuring that the application of predictive models does not compromise human autonomy in clinical decision-making.

To mitigate the risks associated with utilizing predictive models, it is advisable to establish a robust monitoring plan designed to verify the accuracy of model outputs over time. The expected level of accuracy should be based on a consensus regarding the unique definition of clinical data by investigators, ensuring that the models can be reliably applied in real-world settings^[^59^]^.

The validation and clinical implementation of predictive models for cancer metastasis necessitate a comprehensive approach that harmonizes rigorous scientific methodology with strict regulatory compliance. By addressing these critical aspects, researchers and clinicians can collaborate effectively to develop reliable and effective tools aimed at improving patient outcomes in oncology. As the landscape of cancer treatment continues to evolve, the integration of predictive models into clinical practice will be pivotal in advancing personalized medicine and enhancing the overall effectiveness of cancer therapies^[^60^]^. Through continuous collaboration between researchers, clinicians, and regulatory authorities, the ultimate goal of better patient outcomes can be achieved, paving the way for a more informed and targeted approach to cancer treatment.

## Emerging technologies and future directions

Emerging technologies are set to revolutionize the field of predictive modeling for cancer metastasis, offering new possibilities to improve patient outcomes and refine our understanding of cancer progression. One of the most promising developments is the use of liquid biopsies, a minimally invasive technique that allows for the repeated sampling of tumor cells and tumor DNA circulating in the blood. This approach provides key insights into cancer detection and treatment response, enabling clinicians to monitor disease progression more effectively^[^60^]^. Circulating tumor cells (CTCs), which play a critical role in metastasis formation, have become a primary target of liquid biopsies. Recent research demonstrates the power of this technique in identifying minimal residual disease, sometimes detecting metastatic cancer up to two years earlier than conventional imaging methods^[^61^]^. In cancers like breast, colorectal and prostate, the enumeration and molecular analysis of CTCs have proven highly effective for assessing prognosis, with higher CTC counts consistently associated with poorer outcomes. The dynamic nature of CTC counts also makes them valuable for tracking disease status, offering real-time data on treatment response or cancer recurrence.

Simultaneously, AI and ML are transforming cancer research and clinical applications by enabling the analysis of large, complex genomic datasets. These technologies facilitate the identification of predictive biomarkers, advancing our ability to build accurate prognostic models. AI-powered algorithms can process vast amounts of data quickly and efficiently, uncovering patterns and insights that would be difficult to discern through traditional methods. The integration of eXplainable AI (XAI) takes this a step further by improving the transparency and interpretability of AI-driven models. XAI techniques allow researchers and clinicians to understand the decision-making processes within machine learning algorithms, boosting the reliability of these tools in critical medical applications^[^62^]^. By elucidating the mechanisms behind metastasis and identifying key genomic biomarkers, XAI holds the potential to propel transformative advances in cancer research, leading to more tailored treatment approaches.

Another cutting-edge technology, single-cell sequencing, has dramatically enhanced our understanding of the genetic and cellular complexity involved in tumor progression. Unlike traditional sequencing methods that analyze bulk tissue samples, single-cell sequencing allows for the examination of individual cells, revealing the heterogeneity within a tumor^[^63^]^. This is particularly important in cancer research, where intra-tumor heterogeneity influences clonal evolution, invasion, metastasis, and treatment response. Single-cell RNA sequencing (scRNA-seq) and other RNA-based methods have enabled the identification of specific cell types, gene expression patterns and signaling pathways involved in cancer development. The application of this technology extends to analyzing the tumor microenvironment (TME), revealing critical insights into the immune landscape of cancers and the differentiation routes of immune cells. This has led to a more nuanced understanding of how the TME contributes to tumor growth and metastasis, providing new avenues for therapeutic intervention^[^64^]^.

As these emerging technologies continue to evolve, they offer great promise for advancing cancer research and improving clinical outcomes. Liquid biopsies, AI and single-cell sequencing are likely to converge, enabling more comprehensive and personalized approaches to cancer treatment. The integration of these technologies into predictive modeling for cancer metastasis will not only enhance the accuracy of early detection but also lead to more individualized and effective treatment strategies, ultimately transforming the future of cancer care^[^65^]^.

## Challenges and limitations

While predictive modeling for cancer metastasis holds immense promise, it faces several challenges and limitations that must be addressed to ensure its efficacy in clinical settings. One of the foremost issues is data quality and harmonization. Data used in predictive models often come from diverse sources which can lead to inconsistencies and compatibility issues. To overcome this, data harmonization has become crucial, involving the integration of different datasets to make them comparable. However, this is not just a technical challenge but also a theoretical one, as researchers must reconcile varying definitions and operations of concepts across datasets^[^66^]^. The approach to harmonization can be either stringent, using identical measures across studies, or flexible, transforming datasets into a common format while maintaining inferential equivalence. The decision between retrospective harmonization (applying standards to existing data) and prospective harmonization (applying standards as new data is collected) also significantly impacts data quality and, consequently, the accuracy of the models.

Another major challenge is model interpretability. As ML models grow more complex, their transparency becomes a concern, especially in clinical applications. While accuracy is critical for predictive models, clinicians also need interpretable results to trust these models for decision-making^[^67^]^. Explainability refers to a model’s ability to provide reasons for its behavior, while interpretability is the ease with which a human can understand how the model arrived at its predictions based on input data. In fields like radiation oncology, where data sources are complex and varied, deep learning (DL) models may improve prediction accuracy but often lack the transparency needed for clinical trustworthiness. The ability to interpret a model’s decisions is essential for clinicians to feel confident in using it to guide treatment strategies, making this an area of active research^[^68^]^.

The generalizability of these predictive models across different populations is another significant hurdle. Models are often trained on data from specific demographic groups, healthcare settings or geographic locations, which can limit their applicability to broader populations. This lack of generalizability can lead to biased predictions and exacerbate healthcare disparities, especially in underrepresented communities^[^69^]^. To mitigate this, researchers must ensure that the training data used to develop these models is representative of diverse populations^[^70^]^. Moreover, external validation, where the model is tested on data from populations or settings outside the original dataset, is crucial to assess how well the model can generalize to real-world clinical scenarios.

These challenges underscore the need for ongoing refinement of predictive models for cancer metastasis. Researchers must prioritize data harmonization, interpretability and generalizability to ensure these models not only achieve high accuracy but also gain the trust of clinicians and perform well across diverse patient populations^[^71^]^. Addressing these limitations will be essential to unlocking the full potential of predictive modeling in improving patient outcomes and advancing personalized cancer care^[^90,91^]^.

## Conclusion

The field of predictive modeling for cancer metastasis has seen remarkable progress, paving the way for improved patient care and outcomes. The integration of various data types, including clinical, genomic, and imaging information, has a significant impact on the accuracy and reliability of these models. As research continues to advance, the use of machine learning approaches and emerging technologies like liquid biopsies and single-cell sequencing opens up new possibilities to understand and predict cancer progression. Despite these advancements, challenges remain to be addressed to ensure the widespread adoption and effectiveness of predictive models in clinical settings. These include issues related to data quality, model interpretability and generalizability across diverse populations. Moving forward, ongoing collaboration between researchers, clinicians and regulatory bodies will be crucial to overcome these hurdles and to fully harness the potential of predictive modeling in oncology. This collaborative effort aims to improve early detection, personalize treatment strategies, and ultimately enhance patient outcomes in the fight against cancer metastasis.

## Data Availability

Any datasets generated during and/or analyzed during the current study are publicly available.
